# Temporarily increased stroke rate after Takotsubo syndrome: need for an anticoagulation?

**DOI:** 10.1186/s12872-018-0842-0

**Published:** 2018-06-15

**Authors:** Nadine Abanador-Kamper, Lars Kamper, Judith Wolfertz, Marc Vorpahl, Patrick Haage, Melchior Seyfarth

**Affiliations:** 1 Department of Cardiology, Helios University Hospital Wuppertal, University Witten/Herdecke, Germany; Center for Clinical Medicine Witten/Herdecke University Faculty of Health, Wuppertal, Germany; 2 Department of Diagnostic and Interventional Radiology, Helios University Hospital Wuppertal, University Witten/Herdecke, Germany; Center for Clinical Medicine Witten/Herdecke University Faculty of Health, Wuppertal, Germany

**Keywords:** Takotsubo syndrome, Stroke event, Cardiovascular magnetic resonance imaging

## Abstract

**Background:**

Previous studies have reported slightly higher stroke rates in Takotsubo Syndrome compared to acute myocardial infarction. Our goal was to evaluate the temporal course of stroke rates and left ventricular recovery in patients with Takotsubo Syndrome.

**Methods:**

We retrospectively examined the clinical and imaging data of 72 patients with Takotsubo Syndrome. The data collected came from January 2005 to March 2017. Left ventricular performance was evaluated by cardiovascular magnetic resonance imaging (MRI) in all patients during the acute phase of Takotsubo Syndrome and in a follow-up scan 2 months later. Acute stroke and major adverse clinical events, such as myocardial infarction or recurrence of Takotsubo Syndrome and death, were also determined for each patient at 30 days and 12 months after initial presentation.

**Results:**

The MRI scans performed during the acute phase of Takotsubo Syndrome demonstrated apical ballooning with anterior wall motion dysfunction in 65 (90%) patients. Imaging performed 2 months later demonstrated resolution of this in 97% of those patients. Median left ventricular ejection fraction also significantly increased between both scans (49.5% vs. 64.0%, *P* < 0.001). We observed 9 (12%) events in the study population within 12 months of the initial diagnosis of Takotsubo Syndrome. Stroke had an event rate of 2.8% after 30 days and 4.2% after 12 months.

**Conclusions:**

Apical ballooning was found in the majority of our Takotsubo Syndrome patients on the MRI scans performed at presentation. This finding was subsequently associated with higher than expected stroke rates within 30 days of diagnosis and with rapid recovery of left ventricular function within 2 months of diagnosis. This suggests that rapid improvement in left ventricular morphology and function may facilitate the formation of cardiac emboli and consequently increase stroke rates in Takotsubo Syndrome. Although no guidelines currently exist for the treatment of Takotsubo Syndrome, these results may point to a potential role for temporary oral anticoagulation in high-risk patients. Future studies should examine if stroke rates after Takotsubo Syndrome have been underestimated.

**Electronic supplementary material:**

The online version of this article (10.1186/s12872-018-0842-0) contains supplementary material, which is available to authorized users.

## Background

Large anterior wall myocardial infarction is associated with an increased risk of stroke. [1]. Apical ballooning and temporal anterior wall dysfunction in Takotsubo syndrome (TTS) may also lead to a higher risk for acute cardioembolic complications. Registries reporting long-term outcome data for TTS have found stroke rates of 1–1.7% [2, 3]. The aim of this study was to evaluate the temporal course for the risk of stroke in TTS patients.

## Methods

We evaluated 72 patients with TTS in our tertiary care center from January 2005 until March 2017. The diagnosis was established according to the Mayo Clinic diagnostic criteria [4]. The criteria included reversible left ventricular wall motion abnormalities extending beyond a single coronary territory, absence of significant (> 50%) coronary artery stenosis or plaque rupture, new electrocardiogram abnormalities or elevation of cardiac biomarkers, and absence of myocarditis or late gadolinium enhancement on cardiovascular magnetic resonance imaging (MRI). Patients who were diagnosed with TTS and who had a cardiac MRI performed during the hospital stay (MRI scan < 30 days after admission) were included. Exclusion criteria were history of myocardial infarction, coronary bypass surgery, and congenital heart disease. Acute stroke and major adverse clinical events (MACE), which were defined as a composite of myocardial infarction, recurrence of TTS, or death, were evaluated for each patient after 30 days and after 12 months. All patients with acute coronary syndrome were initially treated according to the current guidelines, which include antiplatelet medication. After confirming the diagnosis of TTS medication was individually adapted. The patients who later had a stroke were not previously treated with anticoagulation due to absence of classical indications.

We performed the contrast-enhanced cardiac MRI using a 1.5 T MRI scanner (Philips; Intera Achieva; Best, Netherlands) in all patients during the acute phase of TTS in order to analyze global and regional left ventricular function and to assess myocardial injury.

The MRI protocol included two-dimensional turbo gradient echo sequences in state of the art steady-state free precession technique. These sequences were performed in short axis, four-chamber, two-chamber and three-chamber views of the left ventricle. For the assessment of myocardial oedema, T2-weighted black blood turbo-spin-echo sequences with fat saturation pre-pulse in short axis and in four-chamber views with slice thickness of 8 mm and gap 0 mm covering the complete left ventricle were performed (echo time 90 ms, flip angle 90°, reconstruction matrix 512). These sequences were previously validated [5–9].

For Late Gadolinium Enhancement (LGE) image acquisition, a three-dimensional inversion-recovery turbo gradient echo sequence of the left and right ventricle was performed in the long and short axis views. LGE images were scanned after an interval of 10–15 min post- injection of 0.2 mmol/kg of gadoteridol (Prohance®, Bracco-Imaging, Konstanz, Germany).

Follow-up MRI scans were performed 2 months after initial presentation to reassess global and regional left ventricular function and myocardial injury.

A commercially available software-tool (CMR/CVI 42, Version 4.0, Circle Cardiovascular Imaging Inc., Calgary, Canada) was used for the cardiac MRI image analysis. Assessment of left ventricular functional parameters included determination of global left ventricular ejection fraction (LVEF), left ventricular end-diastolic volumes and wall motion abnormalities (WMA). The WMA were classified as apical, midventricular, or basal ballooning of the left ventricle. Apical ballooning was specifically defined as WMA of the left ventricular apex, all four apical segments, and a minimum of four affected midventricular segments, which was based on the AHA 17-segment model for the analysis of regional left ventricular function [10]. Midventricular ballooning was defined as WMA of all midventricular segments. Basal ballooning was defined as only WMA of all basal segments.

A region was quantitatively described as having myocardial oedema when the signal intensity threshold was > 2 standard deviations (SD) above that of remote normal myocardium in T2-weighted sequences [9].Myocardial oedema volumes (T2-size) were expressed as percentage of the total left ventricular mass.

LGE was defined as hyperenhancement of myocardium that was ≥5 SD above that of the signal intensity threshold of remote normal myocardium, as previously described [11]. A semi-automated quantification in each short axis slice of the entire left ventricle and right ventricle was used for LGE image analysis. Myocardial scarring volumes (LGE size) were expressed as percentage of the total left or right ventricular mass.

The local ethics committee (University Witten/Herdecke) approved this study and patients gave written informed consent. Patients or their treating physicians were contacted by telephone for the standardized interview used for MACE assessment.

Statistical analysis was performed using STATA/IC 14.2 software (Stat Corp, LP, Texas, USA). Categorical variables are described by frequencies; continuous data are expressed as median with maximum – minimum or interquartile range or with mean and standard deviation. To test if distribution of parameters of the two MRI scans were different Wilcoxon matched-pairs signed-ranks test was used. A two-sided *P* value of less than 0.05 was considered to indicate statistical significance.

## Results

**Fig. 1:**
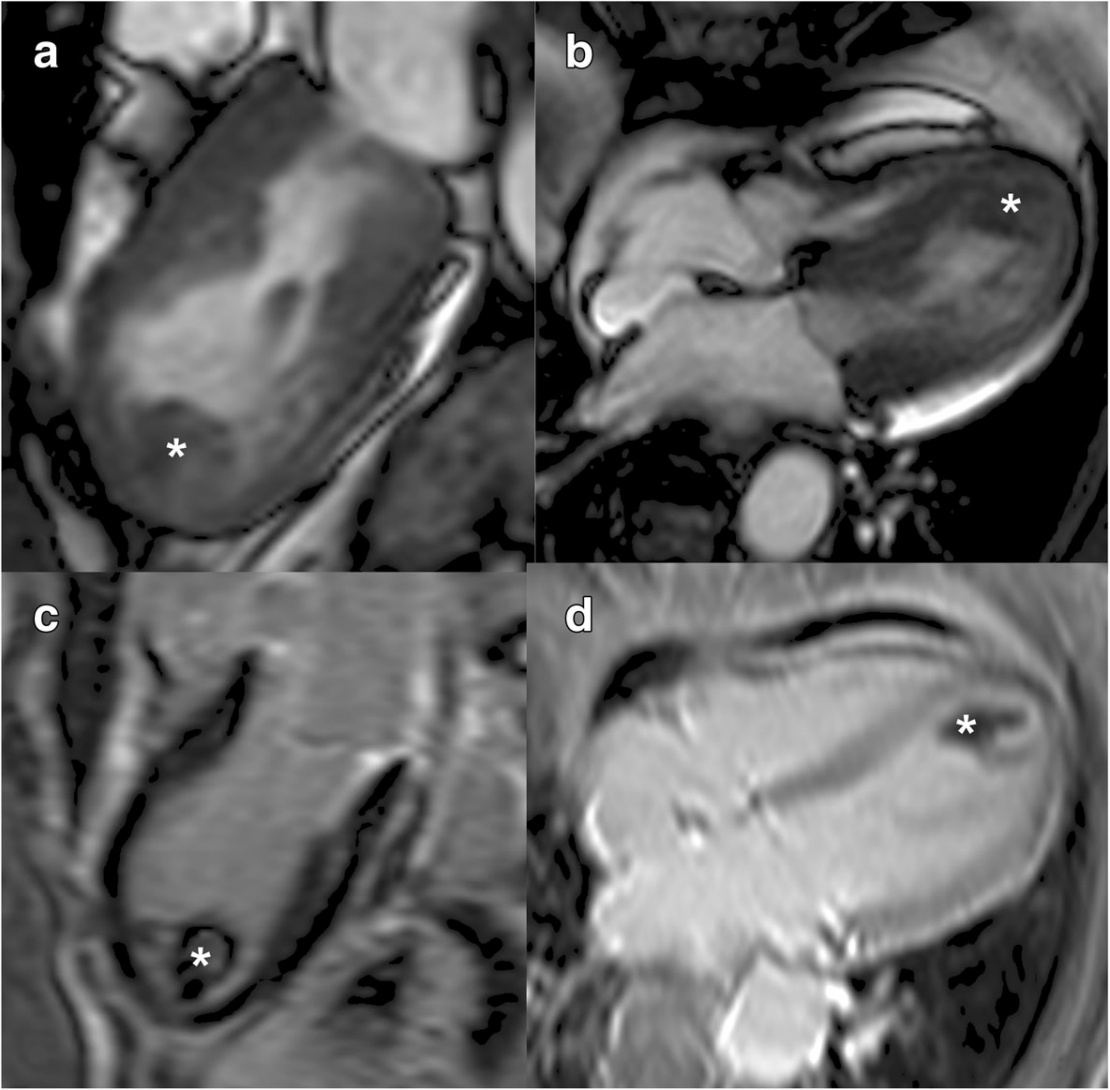
Cardiovascular magnetic resonance imaging of a patient with apical ballooning due to Takotsubo Syndrome. Apical left ventricular thrombus formation (asterisks) in the end-systolic two-chamber view (**a**) and four-chamber view (**b**). Inversion recovery sequence shows a lack of Late Gadolinium Enhancement in the myocardial tissue (**c**) and confirms thrombus in the sequence with long inversion time (**d**)

**Fig. 2:**
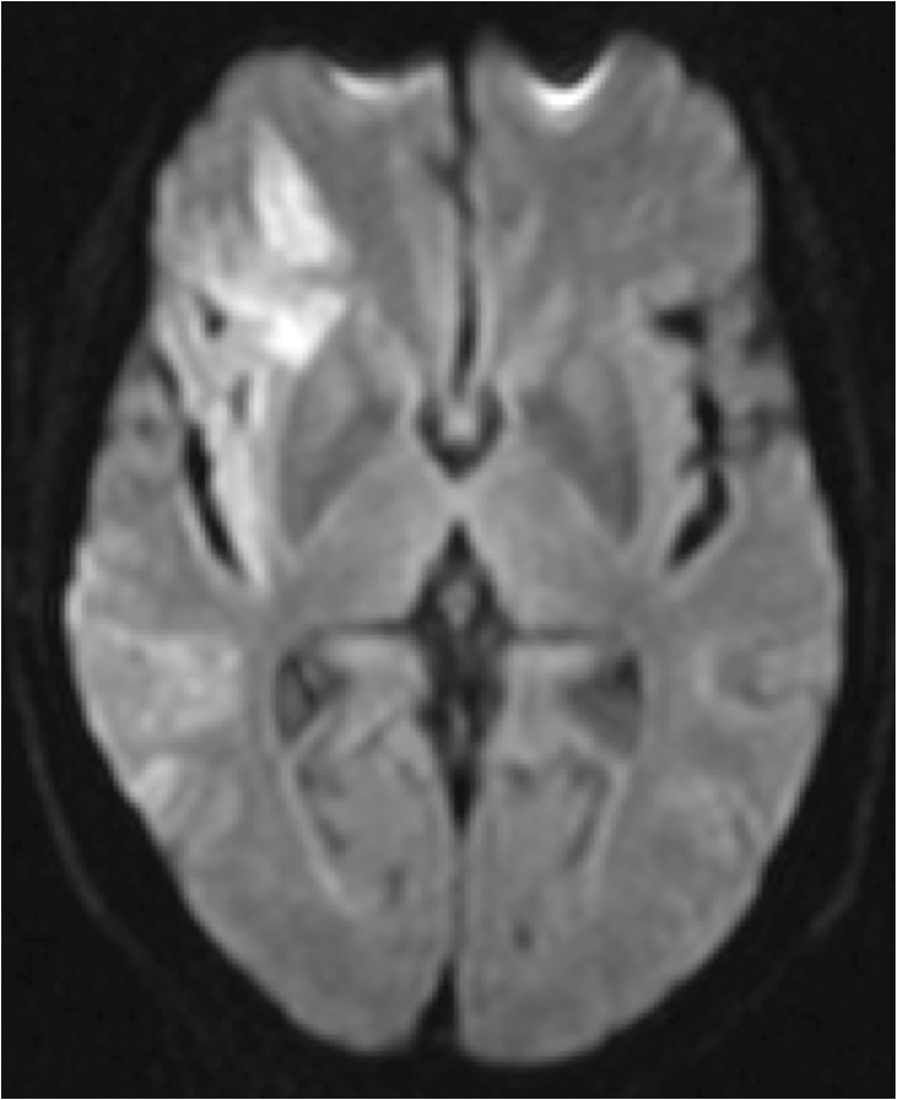
Diffusion-weighted brain MRI of patient who had left ventricular thrombus formation. The scan demonstrates an ischemic stroke in the territory of the right middle cerebral artery


Patients had a mean age of 68.8 ± 17.5 years. The majority were female patients (93%). Patient characteristics are presented in Table 1. We observed 9 (12%) MACE in the study population within 12 months (Table 2). Stroke had an event rate of 2.8% after 30 days and 4.2% after 12 months. Left ventricular thrombus formation (Fig. 1, Additional file 1: Video S1) was found in a patient with acute stroke (Fig. 2). There was no prior anticoagulation in any of the patients who suffered a stroke. The mortality rate was 1.4% after 30 days. After 12 months, three patients had died from non-cardiac events. Two patients had a myocardial infarction or recurrence of TTS (1.4% after 30 days; 2.8% after 12 months).

**Table 1:** Description of study population

Demographic findings	Study population (*n* = 72)
Age (y)	68.8 ± 17.5
Female (n)	67 (93.1%)
Cardiovascular risk factors	
Arterial hypertension	49 (68.1%)
Diabetes mellitus	7 (9.7%)
Current smoking	9 (12.5%)
Hyperlipidemia	20 (27.8%)
Obesity (BMI)	24 (15–39)
Family history for MI	16 (22.2%)

Data is presented as number of patients and percentage. Age, is presented as mean and standard deviation. Body Mass Index is presented as median with minimum and maximum range. *BMI* Body mass index, *MI* myocardial infarction

**Table 2:** Data of MACE

MACE			
	Total	After 30 days	After 12 months
Total events	9 (12%)	4 (5.6%)	9 (12%)
Stroke	3 (4.2%)	2 (2.8%)	3 (4.2%)
Death	4 (5.6%)	1 (1.4%)	4 (5.6%)
MI/TTS	2 (2.8%)	1 (1.4%)	2 (2.8%)

Data is presented as number of patients and percentage. Death events are counted of all causes. *MACE* major adverse clinical events, *MI* myocardial infarction, *TTS* Takotsubo syndrome

Additional file 1: **Video S1**. Steady-state free precession CINEimaging of the cardiac three-chamber view in a patient with Takotsubo Syndrome. Apical ballooning with leftventricular thrombus formation is evident. (AVI 616 kb)


Initial MRI was performed in all patients shortly after presentation with TTS (median: 2d; IQR 1–4), and a follow-up scan was conducted 2 months later (IQR 1.3–2.9) in 63 (88%) patients. Apical ballooning was initially observed in 65 (90%) patients and resolved in 97% of those patients within 2 months (Table 3). Apical ballooning was initially detected in all patients who later suffered a stroke. In all of these cases, stroke events occurred before apical ballooning resolved in the follow-up MRI scan.

**Table 3:** Description of differences between MRI parameters of initial MRI (n = 72) and follow-up scan (*n* = 63)

MRI parameters	MRI I	MRI II	*p*-value
LV function			
LVEF (%)	49.5 (25–73)	64 (49–84)	*< 0.001*
LVEDVI (ml/m^2^)	76.5 (47.0–112.0)	73 (46.0–101.0)	0.198
LV ballooning n (%)			
anterior	65 (90)	2 (3)	*< 0.001*
midventricular	7 (10)	0	0.493
basal	0	0	
Myocardial injury (%)			
T2 Volume	13.5 ± 11.3	0.6 ± 2.4	*< 0.001*
LGE Volume	0.0 ± 0.0	0.0 ± 0.0	

LV ballooning is presented as number of patients and percentage. LVEF, LVEDVI are presented as median with minimum and maximum range. LGE (myocardial scarring) and T2 volumes (myocardial oedema) are presented as mean with standard deviation. *MRI* Cardiovascular magnetic resonance imaging, *AW* anterior wall, *LVEF* left ventricular ejection fraction, *LVEDVI* left ventricular end diastolic volume index, *LV* left ventricular, *LGE* late gadolinium enhancement

*P*-values in italic indicates statistical significance

Median LVEF significantly increased between both scans (49.5% vs. 64.0%, *P* < 0.001). Myocardial oedema decreased significantly between the two MRI scans. LGE was not detected in these TTS patients (Table 3).

## Discussion

Prior studies have reported stroke rates of 0.5–0.9% [1, 12] in acute myocardial infarction and slightly higher stroke rates of 1–1.7% in TTS [2, 3]. A small prospective registry of TTS patients found that 1% of the 209 registry patients suffered a stroke and that all of those with a stroke had progressive LV thrombus formation despite therapeutic doses of heparin or treatment with clopidogrel or aspirin within the acute event [2]. There was a trend towards a lower LVEF in patients with LV thrombus formation and a significantly higher rate of right ventricular involvement. However, quantitative analysis of the regional WMA found in these patients were not precisely performed. Interestingly, the incidence of LV thrombi detection within the first 5 days was relatively high at 3% and might even be an underestimate in this registry due to the fact that not every patient underwent imaging by cardiac MRI. Contrast enhanced MRI is known to be highly sensitive for the detection of LV thrombi in acute myocardial infarction [13].

A larger, prospective registry reported a stroke / transient ischemic attack rate of 1.7% per patient-year. Apical ballooning was found in 81.7% of the enrolled patients; however, precise descriptions of the WMA in the stroke patients were again not supplied. In our study, we found a higher stroke rate (2.8%) in TTS patients within the first 30 days. One explanation for this might be that we also found more apical ballooning with severe anterior WMA. The most common WMA in our study were associated with apical ballooning, which was found in 90% of patients and resolved in 97% of them. Previous studies support this finding; although, the percentage of TTS patients with apical ballooning in these studies was found to be approximately 82% [3, 14]. The higher stroke rate seen in our study may, in part, be secondary to the fact that we also observed more apical ballooning in the TTS patients and that these patients then experienced significant recovery of left ventricular function within 2 months of presentation. Indeed, all of the patients who suffered a stroke had apical ballooning, which resolved completely upon subsequent imaging. Thus, we suggest that rapid changes in left ventricular morphology may facilitate the formation of cardiac emboli and that the risk of stroke after TTS may currently be underestimated.

At this time, there are no standard therapeutic recommendations for TTS available. We propose that oral anticoagulation for 2 months or until recovery of ventricular function might decrease the risk of stroke in TTS.

## Study limitations

A limitation of the study is its retrospective design and the relatively small, single-center cohort. A larger, prospective, multicenter study would be better powered to confirm our hypothesis.

## Conclusions

In conclusion, our findings suggest that apical ballooning with severe anterior WMA followed by a rapid improvement in left ventricular morphology and function may facilitate the formation of cardiac emboli and consequently increase stroke rates in TTS. No guidelines currently exist for the treatment of TTS, but our results point to a potential role for oral anticoagulation in high-risk patients until recovery of left ventricular function.

## Abbreviations

AW: Anterior Wall
IQR: Interquartile range
LGE: Late Gadolinium Enhancement
LV: Left ventricular
LVEF: Left ventricular ejection fraction
MACE: Major adverse clinical events
MRI: Magnetic Resonance Imaging
SD: Standard Deviations
TTS: Takotsubo-Syndrome
WMA: Wall Motion Abnormalities
